# Simultaneous Wasting and Stunting (WaSt), Wasting and Anemia (WaAn) and Wasting, Stunting and Anemia (WaStAn) Among Children 6–59 Months in Karamoja, Uganda

**DOI:** 10.1002/fsn3.71149

**Published:** 2025-11-14

**Authors:** Alex Mokori, Nicholas Kirimi, Amos H. Ndungutse, Zakaria Fusheini, Muzafaru Ssenyondo

**Affiliations:** ^1^ UNICEF Uganda Country Office Kampala Uganda; ^2^ UNICEF Kenya Country Office Nairobi Kenya; ^3^ Independent Scholar; ^4^ UNICEF Moroto Zonal Office Moroto Uganda

**Keywords:** anemia, children, co‐existence, food consumption score, household wealth, Karamoja, maternal education, stunting, wasting

## Abstract

Child malnutrition remains a critical public health challenge in Karamoja, Uganda. This study examined the prevalence and determinants of simultaneous wasting, stunting, and anemia (WaStAn) among children aged 6–59 months using secondary data from the 2022 Food Security and Nutrition Assessment (FSNA). The findings reveal alarmingly high rates of wasting (13.0%), stunting (41.4%), and anemia (55.1%), with 4.6% of children experiencing all three conditions concurrently. Children aged 12–23 months were disproportionately affected, and boys were more vulnerable than girls. The co‐existence of these conditions in the same child reflects multiple, overlapping deprivations—nutritional, environmental, and socioeconomic—that compound risk and elevate mortality. Key predictors of WaStAn included child age, sex, district of residence, household wealth, maternal education, food consumption score, and type of residence. The study underscores the urgent need for integrated, multisectoral interventions that address the root causes of undernutrition and anemia. Recommended actions include early screening and treatment, promotion of optimal infant and young child feeding, micronutrient supplementation, deworming, improved water, sanitation and hygiene (WASH), women's empowerment, and strengthened district‐level capacity to deliver nutrition services at scale. These findings highlight the importance of addressing the triple burden of malnutrition holistically, rather than through siloed approaches, to improve child survival and development outcomes in high‐burden settings like Karamoja.

## Graphical abstract

Simultaneous wasting, stunting, and anemia affect 4.6% of children aged 6–59 months in Karamoja, Uganda. This study highlights the urgent need for integrated, context‐specific strategies to address this critical and under‐recognized burden of child undernutrition.

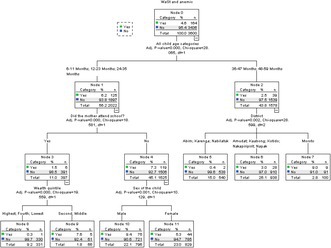

## 1 Introduction

Child undernutrition is a persistent public health concern in low‐ and middle‐income countries, with stunting identified as the predominant condition among children under 5 years (Amare et al. 2019; Bhutta et al. 2020; Ngabo et al. 2023; Madiba et al. 2019). Key indicators for undernutrition include underweight, stunting, and wasting, with stunting having the highest prevalence in low‐ and middle‐income countries (Ngabo et al. 2023; Madiba et al. 2019). Contributors to undernutrition include insufficient food intake, recurrent infections, poor sanitation practices, and low parental education (Ngabo et al. 2023; Madiba et al. 2019). Despite a decline in stunting prevalence in sub‐Saharan Africa (SSA), rates remain above 40%, with persistently high rates in countries like Burundi (Gausman et al. 2022). Implementation of interventions to address childhood undernutrition has been slow, contributing significantly to child mortality in these regions (Akombi et al. 2017). Children aged 1 to 3 years are particularly vulnerable to stunting, underweight, and wasting due to inadequate complementary feeding practices. Globally, 45 million children under five suffer from wasting—14 million of whom are severely affected (Siri and Per 2022)—while 144 million experience stunting and 273 million are anemic, leading to increased mortality risk, impaired development, and heightened susceptibility to infections (Stevens et al. 2022). In Africa, the burden of these conditions exceeds World Health Organization (WHO) thresholds for public health significance (WHO, n.d.). The Karamoja region in Uganda, characterized by high poverty, illiteracy, conflict, and climate shocks, reports elevated rates of wasting (9.6%) and stunting (25.3%) among children aged 6 to 59 months in 2020 (World Food Programme, Uganda Bureau of Statistics, UNICEF, and IBFAN 2020). The prevalence of childhood anemia in this region is notably high at 60.4%, surpassing the national average of 53% (World Food Programme, Uganda Bureau of Statistics, UNICEF, and IBFAN 2020; Uganda Bureau of Statistics and ICF 2018). A study explored risk factors for the coexistence of stunting, underweight, and wasting in sub‐Saharan Africa, revealing variations among countries and regions linked to healthcare access, nutritional practices, beliefs, and governmental health policies (Amadu et al. 2021). Simultaneous wasting and stunting (WaSt) represents a severe form of malnutrition in children, significantly increasing the risks of mortality and morbidity (Briend et al. 2015). WaSt is often associated with chronic exposure to stressors, including food insecurity, infections, poor sanitation, and violence (Khara et al. 2018). Unfortunately, readily available data on the prevalence of WaSt among children aged 6–59 months in the Karamoja region is scarce. However, a study that utilized Demographic and Health Survey (DHS) and Multiple Indicator Cluster Survey (MICS) data from 2000 to 2016 across 84 countries estimated the prevalence of WaSt in Uganda at 2.3%, with regional variations ranging from 0.9% to 4.5% (Khara et al. 2018). Considering the elevated rates of wasting and stunting in the Karamoja region, it is reasonable to infer that the prevalence of WaSt in this region could range between 3% and 5% (Khara et al. 2018), although specific data for Karamoja is not directly available.

Although published evidence remains limited, children with WaSt in Karamoja are likely at high risk of also being anemic. The co‐occurrence of wasting, stunting, and anemia (WaStAn) among children is driven by a complex web of interrelated factors operating across individual, household, community, and national levels (Black et al. 2013). At the individual level, risk factors include age, sex, birth weight, prematurity, genetic predispositions, infections, parasitic infestations, chronic illnesses, suboptimal breastfeeding and complementary feeding practices‐inadequate dietary intake and diversity, micronutrient deficiencies, and immunization status (Briend et al. 2015; Khara et al. 2018; Black et al. 2013). Within households, contributing factors encompass large family size, low income, food insecurity, maternal education and age, poor maternal health and nutrition, maternal anemia and infections, maternal mental health, caregiving practices, paternal involvement, sibling competition, inadequate sanitation and hygiene, and limited access to safe water and healthcare services (WHO 2008). At the community level, poverty, food availability and access, prevailing social norms and beliefs, weak support networks, limited health and nutrition programs, poor WASH infrastructure, and exposure to environmental shocks such as climate change and conflict further exacerbate vulnerability (WHO 2020; UNICEF 2019). National‐level determinants include economic development, food sovereignty, governance of health and nutrition systems, regulatory frameworks for food safety, social protection mechanisms, education and literacy, gender equality, peace and security, and engagement in regional and global initiatives (WHO 2008; Briend et al. 2015). These overlapping deprivations underscore the urgent need for integrated, multisectoral strategies to prevent and manage WaStAn in high‐burden settings like Karamoja.

Yet, there is a distinct gap in addressing the co‐existence of wasting, stunting, and anemia in policy, guidance, programming, and financing, which needs urgent attention (Angood et al. 2016). This is due to limited research in SSA, especially in regions like Karamoja in Uganda, with protracted emergencies and high levels of childhood undernutrition. The research lag underscores the need for more studies utilizing current, representative data to investigate risk factors associated with WaStAn among children under 5 in SSA. Hence, it is important to understand its magnitude to appropriately plan for the prevention, control, and management of children under 5 years with WaStAn in Karamoja region, Uganda. This paper investigates the prevalence and factors associated with WaStAn in children 6 to 59 months in 2022 in Karamoja, Uganda. The aim is to contribute to evidence‐based policies and interventions tailored to the nutritional needs of children in the sub‐region, with potential lessons for other regions within and outside Uganda that are facing similar challenges.

### 1.1 Key Messages

1. A significant proportion (4.6%) of children aged 6–59 months in Karamoja suffer from the simultaneous occurrence of wasting, stunting, and anemia (WaStAn), reflecting multiple, overlapping deprivations that severely increase the risk of child mortality.
2. Children aged 12–23 months are disproportionately affected by WaStAn, underscoring the critical need for early‐life interventions during the complementary feeding window.
3. Male children, those from poorer households, and children of mothers with no formal education are at significantly higher risk of WaStAn, highlighting the need for equity‐focused strategies.
4. District‐level disparities, particularly in Kotido and Moroto, point to the need for localized, context‐specific interventions that address unique vulnerabilities in high‐burden areas.
5. WaStAn is driven by a complex interplay of individual, household, community, and national‐level factors, including food insecurity, poor WASH conditions, maternal health, and limited access to services.
6. Despite the high burden, WaStAn remains under‐recognized in policy, programming, and financing frameworks. There is an urgent need to integrate WaStAn into national nutrition strategies and monitoring systems.
7. Addressing WaStAn requires a holistic, multisectoral approach that includes early screening, treatment, improved feeding practices, micronutrient supplementation, WASH improvements, and women's empowerment.

## 2 Methods

### 2.1 Study Design

This study used secondary data from the district and population‐representative 2022 Karamoja FSNA survey. The original survey computed the sample sizes for each of the nine districts in Karamoja based on the prevalence of malnutrition, food security, and anemia in the Karamoja FSNA 2021. In the calculation of the sample size for anthropometry, a design effect of 1.5 was used across all districts, but the precision varied depending on the prevalence of Global Acute Malnutrition (GAM). A 5% non‐response rate was factored into the determination of the number of children to be assessed during the anthropometric survey.

### 2.2 Sampling Frame and Procedure

The sampling frame used in the nine districts was based on the 2014 Uganda National Population and Housing Census (NPHC) as approved by the Uganda Bureau of Statistics (UBOS). The census frame is a complete list of all census enumeration areas (EAs) created for the 2014 NPHC. In Uganda, an EA is a geographic area that covers an average of 130 households. Currently, Uganda is divided into administrative districts; each district is sub‐divided into sub‐counties, each sub‐county into parishes, and each parish into villages. The sampling frame contains information about the EA location, type of residence (urban or rural), and the estimated number of residential households at the time of the assessment. The adopted sample for FSNA in the Karamoja sub‐region was stratified and selected in two stages (two‐stage cluster sampling methodology). Samples from each district were selected independently in each stratum in two stages. Implicit stratification and proportional allocation were achieved at each of the lower administrative levels by sorting the sampling frame within each sampling stratum before sample selection, according to administrative units at different levels, and by using probability proportional to size selection (PPS).

In the first stage, a probability sample of EAs was randomly and independently selected from each stratum using an updated list of parishes that constitute a district. The sampling frame was sorted by district, sub‐county, parish, village, and EA. A household listing operation was carried out in the EAs, and the resulting lists of households served as the sampling frame for the selection of households in the second stage. Some selected EAs were large, with more than 250 households. To minimize the task of household listing, the large EAs were segmented, and only one segment, with probability proportional to the segment size, was selected for the assessment. Household listing was conducted only in the selected segment, and therefore, the adopted cluster in the survey was either an EA or a segment of an EA.

In the second stage, a fixed number of households was systematically sampled from a newly established household listing for each selected EA, and interviews were conducted in the sampled households. Systematic random sampling methodology was assumed for all districts and was done by ensuring a random start and a calculated sampling interval from the list of village households generated by the Listing Teams. A sampling interval for each village was determined by dividing the total number of verified households by the estimated sample. The first household was determined randomly using the lottery method by drawing a random number within the sampling interval. The interval was thereafter applied across the sampling frame to generate the list of households to be visited in the field. Each team was provided with a list of households to be surveyed daily.

No replacements or changes of the selected households were allowed during the survey implementation stages to minimize bias. If an individual or an entire household was absent, the teams were instructed to return to the household or revisit the absent individual up to two times on the same survey day. If they were unsuccessful after this, the individual or the household was recorded as an absence and not replaced with another household or individual. If the individual or an entire household refused to participate, then it was considered a refusal, and the individual or the household was not replaced with another.

### 2.3 Study Setting

The study took place in the Karamoja sub‐region, which covers an area of 27,528 km and comprises the nine districts of Abim, Amudat, Kaabong, Karenga, Kotido, Moroto, Nabilatuk, Nakapiripirit, and Napak. The region is projected to have a population of 1.4 million in 2022 by UBOS (https://en.wikipedia.org/wiki/Karamoja).

### 2.4 Participants, Recruitment, and Sampling

**FIGURE 1:**
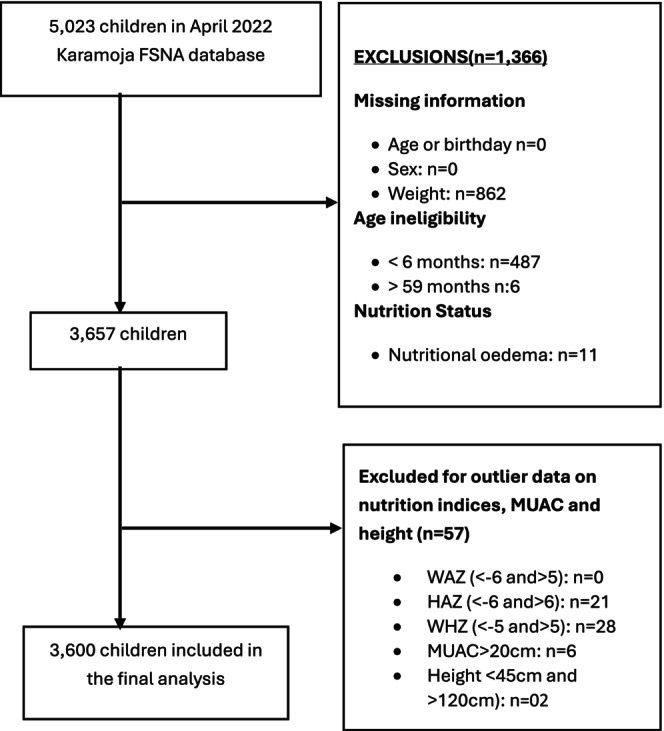
Flow chart showing study participants' selection from April 2022 FSNA database.

The study initially identified 5023 children from the April 2022 Karamoja FSNA database. The sample was refined through the following exclusion steps (Figure 1):

1. Age outliers and nutritional oedema:
  - 493 children under 6 months and above 59 months of age were excluded.
  - 11 with nutritional oedema were excluded.
2. Missing data:
  - 862 children with missing weight were excluded.

This reduced the sample to 3657 children.

3 Outlier nutrition indices, MUAC and height:
  - 57 children were excluded due to incomplete data on the HAZ, WHZ, height and MUAC

After applying these criteria, the final analytical sample consisted of 3600 children, with:

- 50.3% male and 49.7% female
- 33.0% aged 6 to 23 months
- 67.0% aged 24 to 59 months.

### 2.5 Ethical Considerations

The 2022 Karamoja FSNA data set was requested for and shared by the United Nation World Food Programme (WFP) for re‐analysis for this study. No individual identifiers that could jeopardize the respondents were used for the study.

### 2.6 Data Collection

The primary data used for this study were collected using an electronic version of the quantitative data collection questionnaire, which was specifically prepared for use on the Open Data Kit (ODK) platform. The tool was administered through face‐to‐face interviews with mothers, caregivers, and/or household heads in the households of the respondents. To ensure privacy and minimize bias, the interviews were conducted in a private place out of reach of non‐participants. This was done using mobile tablets provided by the WFP. Data collection involved the active participation of the local and civic leaders, who were carefully selected and used as guides to identify households for interviews and provided support during the anthropometric measurements.

The enumerators introduced themselves to the respondents and obtained informed consent before conducting interviews. They briefly explained the purpose of their visit, who funded and supported the study, how data would be collected, the expected duration of the interview, and how the results would be used. Consent was derived from the actual people involved. Care was taken to ensure that information was obtained from a reliable and knowledgeable source, who was an adult member of the household and, in most cases, was the household head or her/his spouse. Field data collection lasted an average of 11 days in the selected districts. A capillary blood sample was taken from the fingertips of children 6–59 months of age and all women 15–49 years old (non‐pregnant, pregnant, and breastfeeding). The hemoglobin (Hb) concentration was recorded to the closest gram per decilitre using the portable HemoCue Hb 201+ Analyzer. Where severe anemia was detected, the child and/or woman was immediately referred for treatment.

The anthropometric tools used included digital Seca weighing scales, height boards and MUAC tapes. Children were weighed using a SECA electronic scale with the precision of 100 g. All children were measured naked following the recommended anthropometric methods. The children's height/length was measured with a precision of 0.1 cm by using height boards. Children were measured lightly dressed with no shoes, hairpieces or barrettes on their head that could interfere with a correct height measurement. Children less than 24 months in height were measured laying down while those of 24 months or above were measured standing. The MUAC was measured in centimeters with a precision of 0.1 cm on the left arm, at the midpoint between the shoulder's tip and the elbow, on a relaxed arm. MUAC was taken only for children between 6 and 59 months of age.

### 2.7 Outcome and Exposure Factors

The outcome of this study was WaStAn, that is, co‐existence of child wasting, stunting, and anemia in the same child. To qualify as WaStAn, the child should have been assessed using anthropometric and biochemical indices and found to have wasting, stunting and anemia. Anemia was defined as having hemoglobin of less than 11 g/dL, wasting as a weight for height *z*‐score (WHZ) less than −2, or MUAC less than 12.5 cm and stunting as a height for age *z*‐score (HAZ) less than −2. WaStAn was defined as having all the parameters for wasting (WHZ < −2 and MUAC < 12.5 cm), stunting (HAZ < −2), and any form of anemia (Hb < 11 g/dL). The exposure variables were broadly categorized into individual child demographic, and nutrition characteristics; maternal demographic and nutrition characteristics; and household socio‐economic characteristics.

### 2.8 Data Management and Analysis

Entry of findings during data collection happened simultaneously at the time of administering the questionnaire, given the use of the mobile tablets based on the ODK software. Data from properly filled questionnaires was submitted online, mainly daily, but was subject to the availability of internet connectivity in the field. All the submitted data was stored on the World Food Programme servers, from where it was accessed for final cleaning and analysis. Data was exported from the database software to Microsoft Excel, SPSS, and ENA for SMART for processing and analysis.

Anthropometric data was exported and analyzed using the WHO child growth software (https://www.who.int/childgrowth/software/en/) for the generation of *z*‐scores, which were used to determine nutritional indicators of WHZ and HAZ. Other data was analyzed using IBM Statistics (SPSS) ver22. Morbidity and other health and sanitation data, such as the prevalence of diseases and conditions occurring 2 weeks prior to the survey, latrine coverage, and health indicators, were reported using descriptive statistics.

Food security data was handled systematically to generate the household wealth index from ownership of household property using principal components analysis. The wealth index was derived from the first principal component, which was then ranked and categorized into quintiles.

The Food Consumption Score (FCS) is a composite indicator used to assess dietary diversity, food frequency, and the relative nutritional importance of food groups, based on a 7‐day recall of foods consumed at the household level. In this study, data to calculate the FCS were collected through structured household interviews using the standardized FSNA questionnaire, which included a 7‐day food frequency module. Households were asked how often they consumed items from eight food groups (plus condiments) during the reference period. The FCS ranges from 0 to 112, with higher scores indicating a greater likelihood of nutritionally adequate food intake. Based on WFP standard cut‐offs, households were categorized into three groups: Poor (0–21), Borderline (21.5–35), and Acceptable (> 35) levels of food consumption. In addition, the Individual Food Diversity Score (IFDS) was used to evaluate the nutritional quality and micronutrient adequacy of individual diets. This score is based on a 24‐h recall of foods consumed by individuals and is calculated by summing the number of different food groups consumed. A higher IFDS reflects a more diverse and potentially more micronutrient‐rich diet. The other facets of food security, such as food sources, expenditures on food, and coping mechanisms, were analyzed largely using descriptive statistics.

Factors associated with malnutrition and food security independently associated with the outcome variables were assessed using binary logistic regression since it was a dichotomous variable (1 = WaStAn, 0 = no WaStAn). Multinomial logistic regression was used for household food consumption diversity (food consumption scores). The variable Food Consumption Group, with 3 categories (1) poor consumption, (2) borderline consumption, and (3) acceptable consumption, was used. The category for acceptable food consumption was used as a reference category in the multinomial logistic regression model. The covariates modeled included household socio‐economic status, mother's education, health and sanitation practices, and morbidity factors, and history of crop cultivation in the case of the food security models.

Frequency tabulation was used to describe the demographics and other study population variables of importance. Measures for central tendency and dispersion (means and standard error of means) were used to describe the variables of importance. Mean and standard error of means (SEM) were used for continuous variables and percentages for categorical variables. One‐way ANOVA was used to determine the mean variation in hemoglobin of children 6–23 months in 2006 and 2011. All data management and statistical analysis were performed with STATA/MP version 11.00 (2007; Stata Corporation, College Station, TX) and IBM SPSS 22. All the statistics were considered significant at a *p*‐value of less than 5%.

### 2.9 Statistical Methodology

Non‐parametric statistical classification tree modeling methodologies, as described in Kajungu's research, were applied for the analysis of data used in this study (Kajungu et al. 2012). The classification and regression tree (CART) analysis was used to investigate direct and indirect measures of predictors of the risk of childhood anemia in children 6–23 months in Uganda. Kajungu and colleagues presented four main reasons for the use of the CART technique over other multivariate analytical methodologies: (1) contrary to classical regression that uses linear combinations, this method does not require the data to be linear or additive; (2) classification tree analysis does not require predefining possible interactions between factors; the resulting classification trees accommodate intuitively more flexible relationships among variables, missing covariate values, multi‐collinearity, and outliers; (3) when values for some predictive factors are missing, they can be estimated using other predictor (“surrogate”) variables, permitting the use of incomplete data sets when generating regression trees; (4) it allows for the calculation of the overall discriminatory power, or relative importance, of each explanatory variable.

### 2.10 Classification Tree Modeling

To gain more insight into factors related to the dependent variables of simultaneous wasting, stunting, and anemia among children 6–59 months in Karamoja in 2022, the binary classification tree modeling methodology, as applied by Kajungu, was used (Kajungu et al. 2012).

### 2.11 Ranking Predictor Variables by Relative Importance

The classification tree analysis aims at developing a simple tree structure for predicting data, resulting in relatively few variables that appear explicitly as splitters, a result that may suggest that the other variables are not important in understanding or predicting the dependent/outcome variable (Kajungu et al. 2012). These researchers indicated that unlike a linear regression model, a variable in a classification tree model can be considered highly important even if it never appears as a node splitter. Because the method keeps track of “surrogate” splits in the tree‐growing process, the contribution a variable can make in prediction is not determined only by primary splits. To calculate a variable importance score, the classification tree analysis method looks at the improvement measure attributable to each variable in its role as either a primary or a surrogate splitter. The values of all these improvements are summed over each node and totaled and are then scaled relative to the best‐performing variable. The variable with the highest sum of improvements is scored 100, and all other variables will have decreasing lower scores. The importance score measures a variable's ability to perform in a specific tree of a specific size either as a primary splitter or as a “surrogate” splitter. The relative importance ranking of variables tends to change dramatically when comparing trees of substantially different sizes. Therefore, the importance scores (rankings) are relative to a given tree structure and should not be interpreted as the absolute information value of a variable. The TREE command in IBM SPSS Statistics version 20 was used to generate the classification trees showing the classification rules generated through recursive partitioning and relative variable importance.

## 3 Results

### 3.1 Prevalence of Child Wasting, Stunting and Anemia Among Children 6–59 Months

The findings showed that 13.0% of the children 6–59 months in the sample had wasting (WHZ), 41.4% were stunted, 9.8% were wasted based on MUAC, and 17.4% had combined GAM (wasting by either WHZ or MUAC). More than half of the children 6–59 months (55.1%) were anemic, with 29.5% and 23.9% being severely and moderately anemic, respectively (Table 1).

**TABLE 1:** Nutritional status among children 6–59 months in Karamoja in 2022.

Nutritional status	Wasting (WHZ)	Wasting (MUAC)	Combined GAM	Stunting (HAZ)	Anemia (Hb)
	*n* (%)	*n* (%)	*n* (%)	*n* (%)	*n* (%)
Not malnourished	3132 (87.0)	3246 (90.2)	2975 (82.6)	2111 (58.6)	1615 (44.9)
Malnourished	468 (13.0)	354 (9.8)	625 (17.4)	1489 (41.4)	1985 (55.1)
Moderately malnourished	369 (10.3)	274 (7.6)	606 (16.8)	775 (21.5)	859 (23.9)
Severely malnourished	99 (2.8)	80 (2.2)	150 (4.2)	714 (19.8)	1062 (29.5)

### 3.2 Nutritional Status Among Children 6–59 Months by Age Group

The findings from the cross‐tabulation analysis revealed the existence of a statistically significant association between the age group and all the nutritional statuses of wasting, stunting, and anemia (*p* < 0.001) (Table 2). The younger children (12–23 months) had a higher prevalence of malnutrition than the older ones. However, it seems the risk of child wasting, indicated by WHZ, decreased with the age of the children, with the highest prevalence being in the age group of 12–23 months (17.1%) and decreasing in the older age groups.

**TABLE 2:** Nutritional status among children 6–59 months by age group.

Age group (months)	*n*	Wasting (WHZ)	Stunting	Wasting (MUAC)	Combined GAM	Anemia
		*n* (%)	*n* (%)	*n* (%)	*n* (%)	*n* (%)
6–11	385	64 (16.6)	83 (21.3)	53 (13.8)	86 (22.3)	249 (6.9)
12–23	803	137 (17.1)	365 (45.5)	142 (17.7)	199 (24.8)	478 (13.3)
24–35	834	112 (13.4)	407 (48.8)	88 (10.6)	149 (17.9)	452 (12.6)
36–47	900	89 (9.9)	374 (41.6)	58 (6.4)	119 (13.2)	445 (12.4)
48–59	678	66 (9.7)	261 (38.5)	13 (1.9)	72 (10.6)	361 (10.0)
Total	3600	468 (13.0)	148 (41.4)	354 (9.8)	625 (17.4)	1985 (55.1)

### 3.3 Prevalence of WaStAn Among Children 6 to 59 Months in Karamoja

As illustrated in Table 3, about 1 in 10 children aged 6–59 months (7.1%) in Karamoja have WaSt, with the highest prevalence being among those in the 12–23 months age group (11.5%) and the lowest in those in the 48–59 months age group (3.5%). The co‐existence of stunting and anemia (StAn) is at 11.6%, and the co‐existence of wasting by combined MUAC and anemia is at 10.5%. The prevalence of WaStAn among children 6 to 59 months in Karamoja is 4.6%.

**TABLE 3:** Prevalence of WaSt, StAn, WaStAn, and combined wasting by child, maternal and household factors.

Variables	WaSt		StAn		WaStAn		Combined WastingAn	
	*n* (%)	*p*	*n* (%)	*p*	*n* (%)	*p*	*n* (%)	*p*
Age group (months)		**< 0.001**		**< 0.001**		**< 0.001**		**< 0.001**
6–11	22 (5.7)		30 (0.8)		17 (0.5)		56 (1.6)	
12–23	92 (11.5)		96 (2.7)		59 (1.6)		119 (3.3)	
24–35	75 (9.0)		119 (3.3)		49 (1.4)		89 (2.5)	
36–47	42 (4.7)		99 (2.8)		25 (0.7)		72 (2.0)	
48–59	24 (3.5)		73 (2.0)		14 (0.4)		43 (1.2)	
Sex		**< 0.001**		**0.012**		**0.001**		**0.006**
Male	165 (4.6)		234 (6.5)		104 (2.9)		216 (6.0)	
Female	90 (2.5)		183 (5.1)		60 (1.7)		163 (4.5)	
District		**< 0.001**		**< 0.001**		**< 0.001**		**< 0.001**
Abim	9 (0.2)		33 (0.9)		3 (0.1)		14 (0.4)	
Amudat	20 (0.6)		48 (1.3)		16 (0.4)		49 (1.4)	
Kaabong	32 (0.9)		39 (1.1)		12 (0.3)		44 (1.1)	
Kotido	52 (1.4)		52 (1.4)		44 (1.2)		96 (2.7)	
Moroto	40 (1.1)		46 (1.3)		22 (0.6)		39 (1.1)	
Nakapiripirit	24 (0.7)		54 (1.5)		19 (0.5)		34 (0.9)	
Napak	35 (1.0)		66 (1.8)		21 (0.6)		44 (1.2)	
Nabilatuk	22 (0.6)		45 (1.2)		14 (0.4)		38 (1.1)	
Karenga	22 (0.6)		34 (0.9)		13 (0.4)		24 (0.7)	
Type of residence		0.522		**0.006**		0.691		0.731
Urban	35 (1.0)		44 (1.2)		23 (0.6)		55 (1.5)	
Rural	220 (6.1)		373 (10.4)		141 (3.9)		324 (9.0)	
Polygamous marriage		0.802		0.795		0.939		0.575
No	152 (4.2)		243 (6.8)		97 (2.7)		218 (6.1)	
Yes	103 (2.9)		174 (4.8)		67 (1.9)		161 (4.5)	
Wealth		< **0.001**		**< 0.001**		**< 0.001**		**< 0.001**
Zero	25 (0.7)		46 (1.3)		15 (0.4)		38 (1.1)	
Lowest	58 (1.6)		86 (2.4)		41 (1.1)		90 (2.5)	
Second	57 (1.6)		85 (2.4)		44 (1.2)		90 (2.5)	
Middle	57 (1.6)		81 (2.2)		36 (1.0)		75 (2.1)	
Fourth	44 (1.2)		64 (1.8)		21 (0.6)		59 (1.6)	
Highest	14 (0.4)		55 (1.5)		7 (0.2)		27 (0.8)	
Mother attending school		< **0.001**		**0.034**		< **0.001**		< **0.001**
No	235 (6.5)		358 (9.9)		156 (4.3)		352 (9.8)	
Yes	20 (0.6)		59 (1.6)		8 (0.2)		27 (0.8)	
Food consumption score		**0.023**		0.230		0.800		0.662
Zero	25 (0.7)		46 (1.3)		15 (0.4)		22 (0.6)	
Acceptable	104 (2.9)		212 (5.9)		78 (2.2)		108 (3.0)	
Borderline	104 (2.9)		131 (3.6)		63 (1.8)		91 (2.5)	
Poor	22 (0.6)		28 (0.8)		8 (0.2)		13 (0.4)	
Total	**255 (7.1)**		**417 (11.6)**		**164 (4.6)**		**379 (10.5)**	

*Note:* Bold values are statistically significant.

The cross‐tabulations indicated a significant association between WaSt and child age (*p* < 0.001), sex (*p* < 0.001), district (*p* < 0.001), wealth index (*p* < 0.001), maternal education (*p* < 0.001), and food consumption score (*p* = 0.023). WaStAn was significantly associated with child age (*p* < 0.001), sex (*p* < 0.001), district (*p* < 0.001), wealth level (*p* < 0.001), and maternal education (*p* < 0.001). StAn had a significant association with child age (*p* < 0.001), sex (*p* = 0.012), district (*p* < 0.001), type of residence (*p* = 0.006), wealth level (*p* = 0.005), and maternal education (*p* < 0.034). Combined WastingAn was associated with child age (*p* < 0.001), sex (*p* = 0.006), district (*p* < 0.001), wealth level (*p* < 0.001), and maternal education (*p* < 0.001). It is remarkable that age group, sex, district, wealth index, and maternal education were significantly associated with WaSt, WaStAn, StAn, and Combined WastingAn. Type of residence and the food consumption score were only associated with StAn (*p* = 0.006) and WaSt (*p* = 0.023), respectively.

The children aged 12–23 months had the highest prevalence of WaSt (11.5%), WaStAn (1.6%), and combined WaStAn (3.3%), while those aged 48–59 months had the lowest prevalence. However, StAn was highest among the children aged 24–35 months (3.3%) and lowest among those in the age group 6–11 months (0.8%). Across all the nutritional status indices, the male child had a higher prevalence of WaStAn compared to their female counterparts.

Kotido district had the highest prevalence of WaSt (1.4%), WaStAn (1.2%), and combined WastingAn (2.7%), followed by Moroto district. StAn was only significantly associated with the area of residence. The children living in rural areas had a significantly higher prevalence of StAn (10.4%) compared to those in urban areas (1.2%).

### 3.4 Predicators of WaSt Among Children 6 to 59 Months in Karamoja

Using the variables in Table 3 to fit the model, the most important predictors of WaSt in Karamoja were investigated. The most important predictor of WaSt among children 6 to 59 months in Karamoja in 2022 was the district of origin, with discriminatory power of 100.0%. This was followed by wealth quintile with a power of 75.3%, child age (62.2%), food consumption score (35.6%), and sex of the child (34.1%) (Table 4).

**TABLE 4:** Ranking of predictors of WaSt among children 6–59 months by their overall discriminatory power.

Independent variable	Power
District	100.0
Wealth quintile	76.7
Child age	63.5
Sex of the child	40.0
Food consumption score	35.7
Maternal education	33.7

**FIGURE 2:**
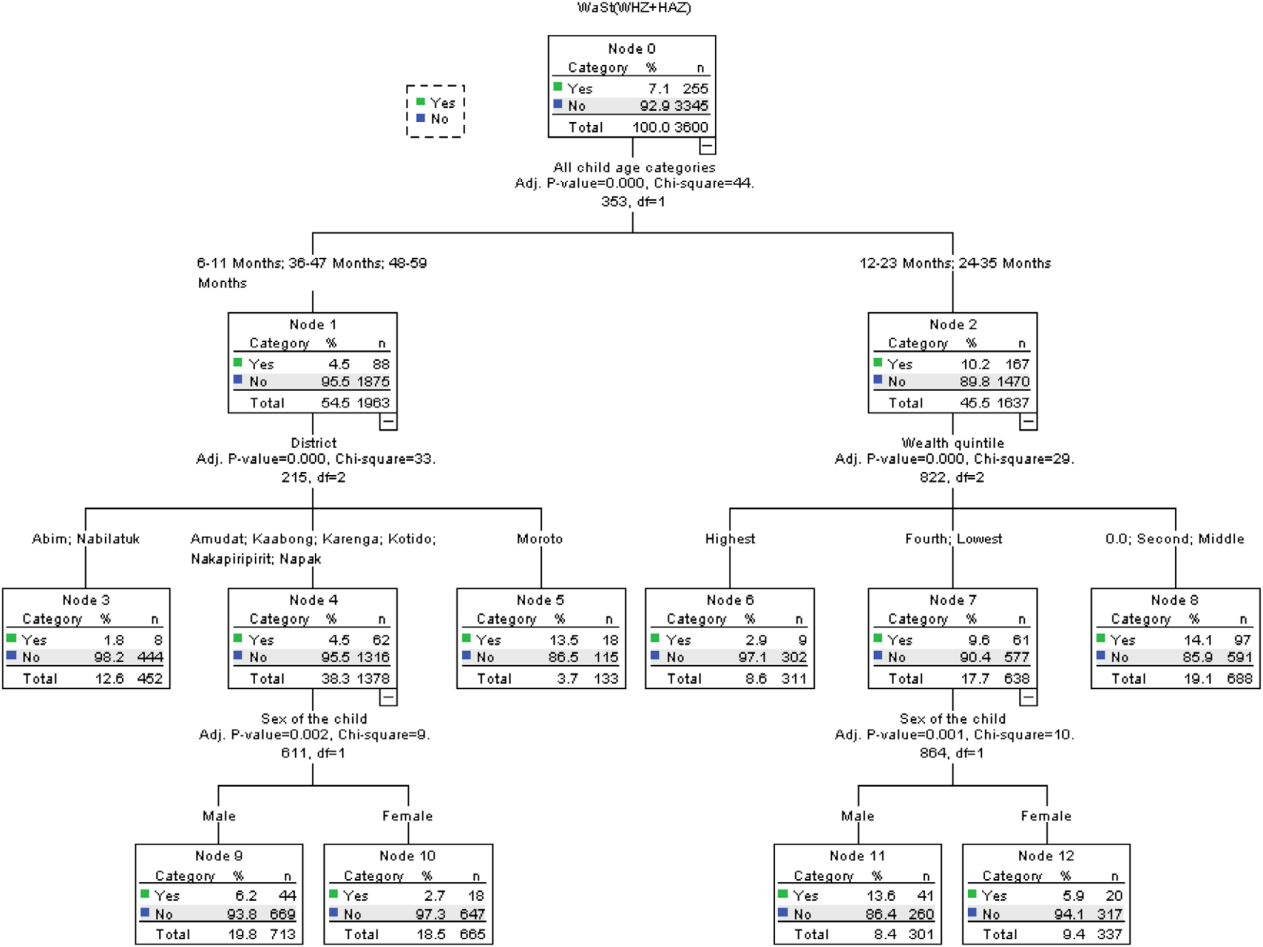
Predictors of WaSt among children 6–59 months by their overall discriminatory power.

Children aged 12–23 and 24–35 months had the highest prevalence of WaSt (10.2%) compared to those aged 6–11, 36–47, and 48–59 months (4.5%). Among the children aged 12–35 months, those from households with wealth index zero, second, and middle had the highest prevalence of WaSt (14.1%), followed by those from the lowest and fourth (9.6%) and the highest (2.9%). Among the children in the fourth and lowest household index, the male had a significantly higher prevalence of WaSt (13.6%) than the female ones (5.9%). Among the children aged 6–11 and 36–59 months, those from Moroto had a statistically significantly higher prevalence of WaSt (13.5%) than the other districts. This was closely followed by those from Amudat, Kaabong, Karenga, Kotido, Nakapiripirit, and Napak (4.5%), and Abim and Nabilatuk had the least prevalence of WaSt (1.8%). Similarly, the male children from Amudat, Kaabong, Karenga, Kotido, Nakapiripirit, and Napak had a higher prevalence of WaSt (6.2%) than the female (2.75%) (Figure 2).

### 3.5 Predicators for WaStAn Among Children 6 to 59 Months in Karamoja

Using the variables in Table 5 to fit the model, the most important predictors of WaStAn were investigated. The most important predictor of WaStAn among children 6 to 59 months in Karamoja in 2022 was the district of origin, with discriminatory power of 100.0%. This was followed by wealth quintile with a power of 61.9%, child age (54.4%), and maternal education (28.5%).

**TABLE 5:** Ranking of predictors of WaStAn among children 6–59 months by their overall discriminatory power.

Independent variable	Power
District	100.0
Wealth quintile	61.9
Child age	54.2
Maternal education	28.5

**FIGURE 3:**
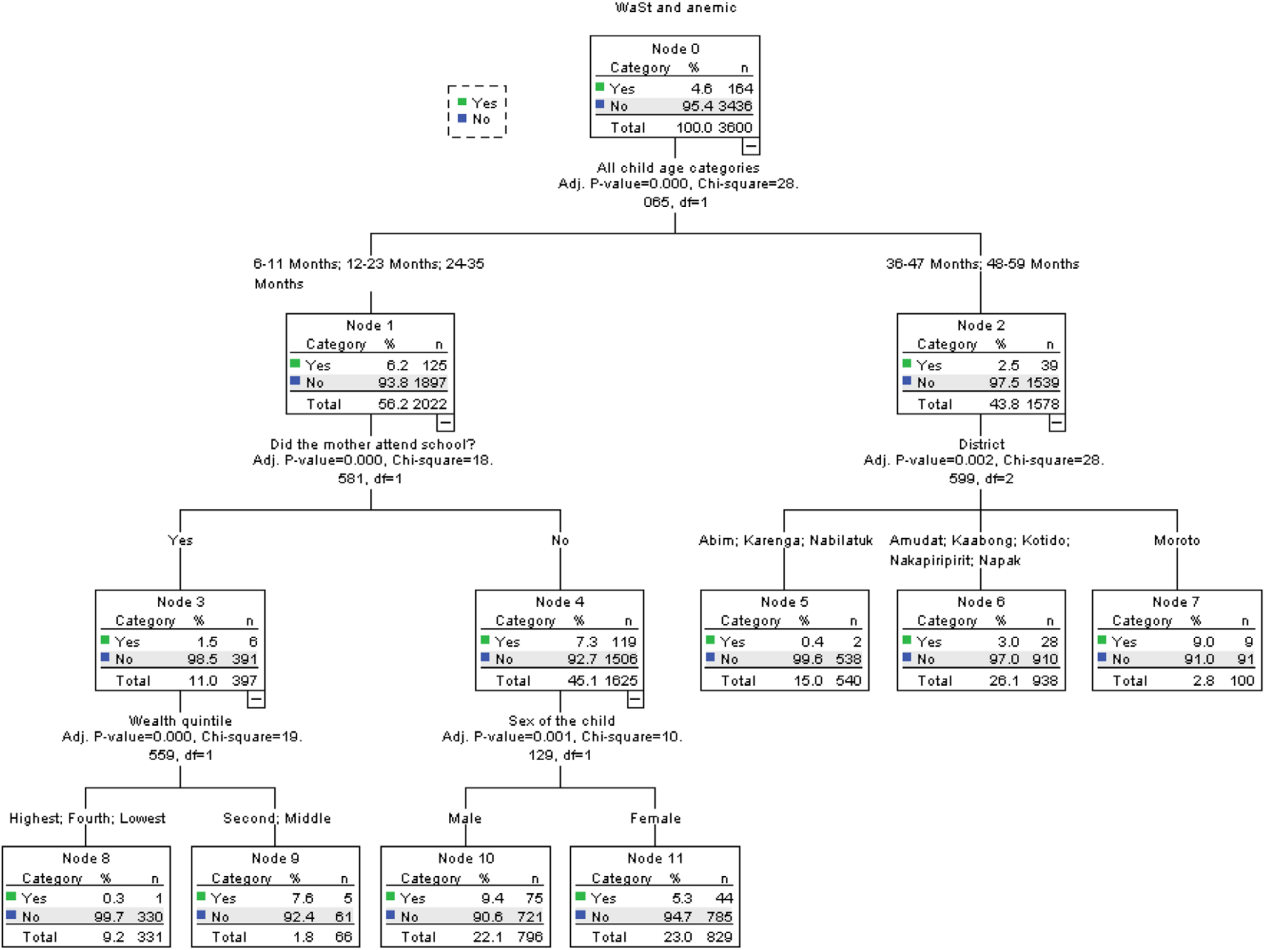
Predictors of WaSt among children 6–59 months by their overall discriminatory power.

Younger children aged 6–35 months tended to have WaStAn (6.2%) compared to the older ones (36–59 months) with a prevalence of 2.5%. The youngest children whose mothers did not have any formal education tended to be more affected by WaStAn (7.3%) than those with mothers with formal education (1.5%). Furthermore, the male children whose mothers did not have formal education were at a higher risk of WaStAn (9.4%) than the female children (5.3%). Among the older children aged 36–59 months, those from Moroto had the highest risk of having WaStAn (9.0%) compared to those from Amudat, Kaabong, Kotido, Nakapiripirit, and Napak (3.0%) or Abim, Karenga, and Nabilatuk (0.4%). The Figure 3 below provides details of these findings.

### 3.6 Predicators for StAn Among Children 6 to 59 Months in Karamoja

Table 6 below shows the relative importance of each of the dependent variables. The district of origin (100%), age of household head (54.7%), and total livestock owned (49.4%) were the most important predictors of StAn in Karamoja. These were followed by the level of education of the household head (41.3%), household wealth quintile (41.2%), marital status of the household head (29.7%), type of residence (28.1%), and child age (27.9%).

**TABLE 6:** Ranking of predictors of StAn among children 6–59 months by their overall discriminatory power.

Independent variable	Normalized importance
District	100.0
Age of household head	54.7
Total livestock owned	49.4
Highest level of school attended by the household head	41.3
Wealth quintile	41.2
Marital status of household head	29.7
Residence	28.1
All child age categories	27.9
Education attendance by household head	25.9
Sex of the child	18.9
Food consumption score	9.3
Did the mother attend school?	7.9

**FIGURE 4:**
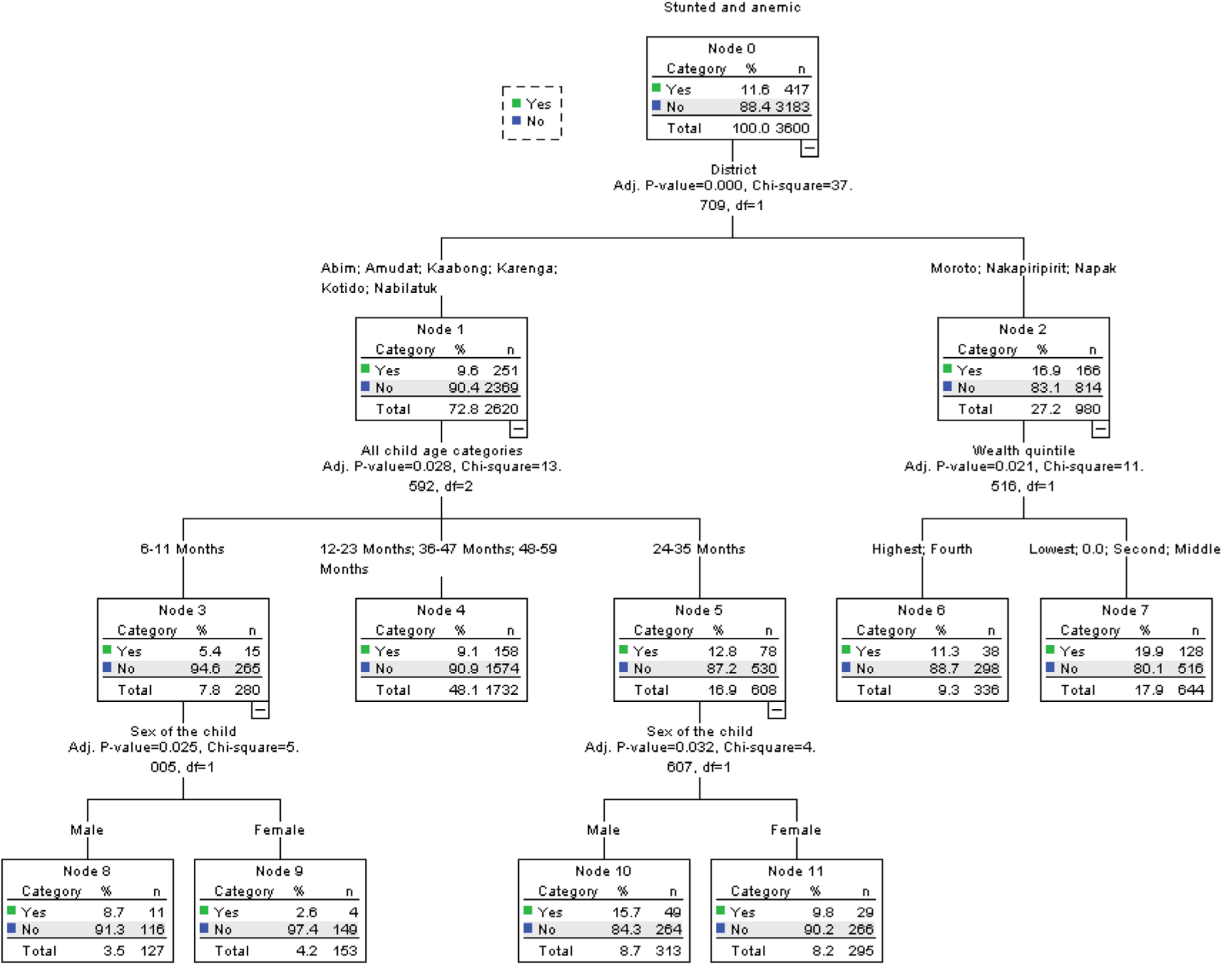
Predictors of StAn among children 6–59 months by their overall discriminatory power.

The district was the most important predictor of StAn among children 6 to 59 months in Karamoja in 2022. Children from the districts of Moroto, Nakapiripirit, and Napak had a higher risk of StAn (16.9%) compared to those from Abim, Amudat, Kaabong, Karenga, Kotido, and Nabilatuk (9.6%). The children in the lowest wealth index in Moroto, Nakapiripirit, and Napak had a significantly higher risk of StAn (19.9%) compared to those from the highest wealth index (11.3%). Within the children from Abim, Amudat, Kaabong, Karenga, Kotido, and Nabilatuk, those in the age group of 24–35 months had the highest risk of StAn (12.8%) compared to those in 6–11 months (5.4%), and 12–23, 36–47, and 48–59 months (9.1%). Of those in the age group 24–35 months, the male was at a higher risk of StAn (15.7%) than the female (9.8%). This is similarly the case with the children in the age group of 6–11 months, in which the prevalence of StAn is 8.7% compared to 2.6% among the female (Figure 4).

## 4 Discussion

The findings from this study highlight the critical need for context‐specific interventions to address the complex interplay of factors contributing to child malnutrition in Karamoja. The high prevalence of WaSt, StAn, and WaStAn among children aged 6 to 59 months underscores the urgency for integrated, multisectoral strategies that simultaneously improve health services, promote diverse and resilient food systems, and mitigate seasonal vulnerabilities. Future research should explore the causal relationships between identified factors and WaStAn and investigate specific dietary deficiencies contributing to WaStAn in Karamoja.

This is the first study to use secondary data from a representative population sample of children 6 to 59 months from all the nine districts of Karamoja, and in Uganda, to document evidence of the prevalence of the simultaneous existence of child wasting with stunting (WaSt), stunting with anemia (StAn), and wasting, stunting, and anemia (WaStAn). It is also the first study to use TREE analysis to determine the predictors of WaSt, StAn, and WaStAn among children 6 to 59 months in the Karamoja region and in Uganda. The study therefore adds to the body of nutrition and public health literature on the prevalence and associated factors of the simultaneous existence of child wasting, stunting, and anemia (WaSt, StAn, and WaStAn) among children 6 to 59 months in Karamoja to inform program managers, policy makers, and academia of this increasing phenomenon.

The paper confirms the continued existence of a high public health nutrition burden of wasting, stunting, and anemia among children 6 to 59 months in Karamoja. Child wasting at 13% and stunting at 41.4% are very high public health problems in Karamoja. Similarly, childhood anemia (55.1%) is a severe public health problem in the region. The stunting, wasting, and anemia levels in Karamoja are much higher than the national ones. These findings indicate a high prevalence of malnutrition in Karamoja and given that the results are higher than national averages, they highlight the region's unique challenges and urgent need to improve food security and nutrition. In fact, it was earlier reported that malnutrition within the region was increasing, with stunting a public health concern (Karamoja Resilience Unit 2018). This contrasts with what is reported about child wasting and stunting declining over the past few decades globally (Bhutta et al. 2020; Roba and Başdaş 2023; Derso et al. 2017). It shows the dire situation in Karamoja and the need for multiple systems‐based strategies to enhance nutrition service delivery, diets, and practices.

It is no surprise that the simultaneous existence of child wasting, stunting, and anemia among children 6 to 59 months in Karamoja is high. The study reported that 7.1% and 11.6% of the children have WaSt and stunting and StAn, respectively, while 4.6% have WaStAn. It is agreed that children can be concurrently wasted and stunted (Roba and Başdaş 2023). However, this increases their mortality risk (Khara et al. 2018). These findings are not surprising considering the multiplicity of deprivations faced by the children in Karamoja, including chronic food insecurity, inadequate consumption of quality diets, and a high level of malaria, diarrhea, and other common childhood illnesses. It shows that there is a complex interplay of factors contributing to malnutrition in Karamoja. Thus, this myriad of factors could expose the same child to wasting, stunting, and anemia at the same time. The study underscores the need for a holistic approach to preventing, controlling, and managing child wasting, stunting, and anemia in Karamoja.

Whereas there is a myriad of factors contributing to child wasting, stunting, and anemia in Karamoja, the findings from this study show that age, sex, district of residence, wealth index, and maternal education were significantly associated with WaSt, StAn, and WaStAn among children 6 to 59 months. The type of residence and the food consumption score were only associated with StAn and WaSt.

It is evident that the youngest children of the age group 12 to 23 months had the highest prevalence of WaSt (11.5%) and WaStAn (1.6%), while those of age 48 to 59 months had the lowest prevalence. Similar findings were also reported by a recent study which observed that the highest prevalence of wasting occurs in young children aged between 6 and 23 months (Derso et al. 2017). Child age ranked as the third most important predictor of WaSt (62.2%). Collectively, children of age 12–23 and 24–35 months had the highest prevalence of WaSt (10.2%) compared to those of age 6–11, 36–47, and 48–59 months (4.5%). Similarly, child age (54.4%) was the third most important predictor of WaStAn among children 6 to 59 months in Karamoja. Younger children of age 6 to 35 months tended to have WaStAn (6.2%) compared to the older ones (36–59 months) with the prevalence of 2.5%. Child age (27.9%) was the least important predictor of StAn among children 6 to 59 months in the region. Among the children from Abim, Amudat, Kaabong, Karenga, Kotido, and Nabilatuk, those in the age group of 24–35 months had the highest risk of StAn (12.8%) compared to those in 6–11 months (5.4%), and 12–23, 36–47, and 48–59 months (9.1%). Of those in the age group 24–35 months, the male was at a higher risk of StAn (15.7%) than the female (9.8%). This is similarly the case with the children in the age group of 6–11 months, in which the prevalence of StAn is 8.7% compared to 2.6% among the female. An earlier study also confirmed that the prevalence of WaStAn increased in boys (Roba and Başdaş 2023).

StAn was highest among children aged 24–35 months (3.3%) and lowest among those aged 6–11 months (0.8%), aligning with existing evidence that stunting and anemia, both cumulative conditions, manifest more prominently in later stages of early childhood (Odei Obeng‐Amoako et al. 2021). This reflects the known trajectory of undernutrition, where wasting often begins during the transition to complementary feeding (6 to 8 months) and peaks between 9 and 23 months, while stunting and anemia emerge more gradually.

However, the observed decline in the prevalence of WaSt, WaStAn, and StAn after 35 months warrants further research. Existing evidence suggests that children with multiple anthropometric deficits, particularly WaStAn, are at a significantly increased risk of early mortality, which may contribute to lower observed prevalence in older age groups (Khara et al. 2018). Additionally, some studies indicate that nutritional recovery may occur over time due to improved dietary intake, reduced exposure to infections, and enhanced caregiving practices, potentially leading to a reversal of some deficits in later childhood (Bhutta et al. 2020). These patterns highlight the importance of early, targeted interventions and the need for longitudinal research to examine survival and recovery trajectories among children experiencing multiple forms of undernutrition in high‐burden settings such as Karamoja.

Among the children of age 12–35 months, those from households with a wealth index of second and middle had the highest prevalence of WaSt (14.1%), followed by those from the lowest and fourth (9.6%) and the highest (2.9%). In fact, poor wealth status is associated with high levels of WaSt among children aged 6–24 months (Derso et al. 2017). The youngest children whose mothers did not have any formal education tended to be more anemic (7.3%) than those with mothers with formal education (1.5%). This finding is similar to that reported in the Karamoja FSNA report in 2018. The report revealed that there was a strong association between low levels of education among heads of households and mothers with a high prevalence of wasting, stunting, and underweight in children below the age of five (Karamoja Resilience 2018). This is a challenge given that maternal education is considered a contributor to the improvement of children's nutrition (Bhutta et al. 2020; Roba and Başdaş 2023). There is a need for interventions that address the social determinants of malnutrition in Karamoja.

Across all the nutritional status indices, the male child had the highest prevalence compared to their female counterparts. This finding aligns with previous studies where boys exhibited a greater risk of wasting, stunting, and anemia (Roba and Başdaş 2023). While the reasons for this disparity are not fully understood, some studies suggest biological susceptibility among male children to environmental stressors and infections. In other contexts, research has also pointed to potential differences in caregiving or feeding practices, where girls may be preferentially fed or cared for over boys (Odei Obeng‐Amoako et al. 2021). Further research is warranted to explore the underlying causes of these sex‐based differences in nutritional outcomes in Karamoja.

The study also reveals significant differences in malnutrition prevalence across districts in the Karamoja sub‐region. Kotido district had the highest prevalence of WaSt (1.4%), StAn (1.2%), and combined WaStAn (2.7%), followed by Moroto district. StAn was only significantly associated with the area of residence. The children living in rural areas had a significantly higher prevalence of StAn (10.4%) compared to those in urban areas (1.2%). The most important predictor of WaSt among children 6 to 59 months in Karamoja in 2022 was the district of origin, with a discriminatory power of 100.0%. Among the older children 36–59 months, those from Moroto had a higher risk of having WaStAn (9.0%) than those from Amudat, Kaabong, Kotido, Nakapiripirit, and Napak (3.0%) or Abim, Karenga, and Nabilatuk (0.4%). The district of origin (100%), age of household head (54.7%), and total livestock owned (49.4%) were the most important predictors of StAn in Karamoja. The district was the most important predictor of StAn among children 6 to 59 months in Karamoja in 2022. Children from the districts of Moroto, Nakapiripirit, and Napak had a higher risk of StAn (16.9%) compared to those from Abim, Amudat, Kaabong, Karenga, Kotido, and Nabilatuk (9.6%). The children in the lowest wealth index in Moroto, Nakapiripirit, and Napak had a significantly higher risk of StAn (19.9%) compared to those from the highest wealth index (11.3%).

The district‐level differences in malnutrition prevalence likely reflect variations in food access and exposure to shocks. Kotido and Moroto, which had the highest burden, frequently face seasonal food insecurity, limited livelihoods, and recurrent conflict, including cattle raids. In contrast, Abim and Nabilatuk benefit from more stable climates, lower conflict, and greater agricultural potential, contributing to better food access. Uneven coverage of humanitarian and nutrition programs may further explain these disparities. Although food access was not directly assessed, the observed patterns align with documented contextual realities in Karamoja that are well recognized by practitioners.

The observed high simultaneous stunting, wasting, and anemia among children 6–59 months in Karamoja, Uganda, reflects the profound short‐ and long‐term effects of infections, inadequate diets and seasonal food insecurity. The region has a high prevalence of infections such as malaria, diarrhea, pneumonia and worms which contribute to undernutrition by impairing nutrient absorption, increasing metabolic demands, and triggering inflammation that disrupts growth and iron metabolism. These biological stressors are compounded by limited dietary diversity, as many households rely on monotonous diets low in animal‐source foods, fruits, and vegetables, leading to chronic deficiencies in essential nutrients like iron, zinc, and vitamin A. Seasonal food insecurity further exacerbates this burden; during lean periods, food availability and access decline, dietary quality worsens, and disease prevalence rises, particularly due to poor water, sanitation and hygiene conditions. These overlapping factors create a vicious cycle where children weakened by one form of malnutrition become more susceptible to others.

Despite the strengths of this study, including the use of a large, population‐representative dataset and advanced analytical techniques such as classification tree modeling, several limitations must be acknowledged. First, the cross‐sectional design precludes causal inference. Second, the dataset lacked direct measures of household food insecurity, health service access, and disease burden—factors that could further clarify observed disparities. Third, classification tree analysis, while robust in handling complex interactions, may be sensitive to overfitting, limiting generalizability. Fourth, some variables were based on self‐reports and thus may be subject to recall or reporting bias. Finally, although district‐level differences were explored, broader structural factors such as climate variability, conflict dynamics, and programmatic intensity were not systematically measured, constraining the contextual depth of the findings.

Considering the above, future research should explore the causal relationships between identified factors and WaStAn. It should also investigate specific dietary deficiencies contributing to WaStAn in Karamoja.

## 5 Conclusions

This study provides evidence of the simultaneous wasting, stunting, and anemia (WaStAn) among children aged 6–59 months in Karamoja, Uganda. The high prevalence of these conditions—individually and in combination—underlines the urgent need for integrated, multisectoral interventions that address both wasting and chronic nutritional deficiencies and anemia.

Children aged 12–23 months, males, those from poorer households, and children of mothers with low educational attainment are at significantly higher risk. District‐level disparities, particularly in Kotido and Moroto, further emphasize the need for localized, context‐specific responses. The findings also confirm that wasting peaks earlier in life, while stunting and anemia become more prominent in later months, reinforcing the importance of early, age‐specific interventions. The co‐existence of these conditions reflects a complex interplay of biological, socioeconomic, and environmental factors. Addressing this “triple burden” requires holistic programming that moves beyond siloed approaches. Interventions should include early detection for wasting and anemia, improved infant and young child feeding, micronutrient supplementation, WASH and health improvements, and women's empowerment. Policy and programmatic efforts must prioritize vulnerable groups and be tailored to district‐specific contexts. Continued investment in nutrition strategies and their delivery across the different essential systems including health, WASH, social protection and food, is essential to reduce child mortality and improve long‐term development outcomes in Karamoja.

## Author Contributions

**Alex Mokori:** conceptualization (lead), data curation (lead), formal analysis (equal), funding acquisition (supporting), investigation (lead), methodology (lead), project administration (equal), resources (equal), software (equal), supervision (lead), validation (lead), visualization (equal), writing – original draft (lead), writing – review and editing (lead). **Amos H. Ndungutse:** conceptualization (supporting), data curation (supporting), formal analysis (supporting), funding acquisition (supporting), investigation (supporting), methodology (equal), project administration (equal), resources (supporting), software (supporting), supervision (equal), validation (equal), visualization (supporting), writing – original draft (equal), writing – review and editing (supporting). **Nicholas Kirimi:** conceptualization (supporting), data curation (lead), formal analysis (lead), funding acquisition (supporting), investigation (supporting), methodology (supporting), project administration (supporting), resources (supporting), software (supporting), supervision (supporting), validation (supporting), visualization (equal), writing – original draft (supporting), writing – review and editing (supporting). **Zakaria Fusheini:** conceptualization (supporting), data curation (supporting), formal analysis (supporting), funding acquisition (supporting), investigation (supporting), methodology (supporting), project administration (supporting), resources (equal), software (supporting), supervision (lead), validation (equal), visualization (supporting), writing – original draft (supporting), writing – review and editing (supporting). **Muzafaru Ssenyondo:** conceptualization (supporting), data curation (supporting), formal analysis (supporting), funding acquisition (supporting), investigation (supporting), methodology (supporting), project administration (supporting), resources (supporting), software (supporting), supervision (supporting), validation (supporting), visualization (supporting), writing – original draft (supporting), writing – review and editing (supporting).

## Ethics Statement

This research study did not require an Institutional Review Board Statement because secondary data shared by the World Food Programme was reviewed and analyzed by the authors of this research paper. There was no direct involvement or contact with human subjects, and therefore the study did not violate the Declaration of Helsinki code; thus, no protocol code or date of approval is required in our paper submission.

## Consent

In this study, there was no direct contact with participating patients. The authors believe there was no need for informed consent to be obtained from the participating patients.

## Conflicts of Interest

The authors declare no conflicts of interest.

## Data Availability

The data set shared by WFP was analyzed for this study and is available upon request.
